# *Flow Index*: a novel, non-invasive, continuous, quantitative method to evaluate patient inspiratory effort during pressure support ventilation

**DOI:** 10.1186/s13054-021-03624-3

**Published:** 2021-06-07

**Authors:** Filippo Albani, Luigi Pisani, Gianni Ciabatti, Federica Fusina, Barbara Buizza, Anna Granato, Valeria Lippolis, Eros Aniballi, Francesco Murgolo, Antonio Rosano, Nicola Latronico, Massimo Antonelli, Salvatore Grasso, Giuseppe Natalini

**Affiliations:** 1 Department of Anesthesia and Intensive Care, Fondazione Poliambulanza, Brescia, Italy; 2 Department of Anesthesia and Intensive Care, Miulli Regional Hospital, Acquaviva Delle Fonti, Bari, Italy; 3 Mahidol Oxford Clinical Research Unit (MORU), Bangkok, Thailand; 4 Department of Anesthesiology, Neurointensive Care Unit, Azienda Ospedaliera Universitaria Careggi, Firenze, Italy; 5 Department of Anesthesia and Intensive Care, Spedali Civili, Brescia, Italy; 6 Department of Anesthesia and Intensive Care, Mater Dei Hospital, Bari, Italy; 7 Department of Anesthesia, I.R.C.C.S. MultiMedica, Sesto San Giovanni, Milano, Italy; 8 Department of Emergency and Organ Transplantation, University of Bari, Bari, Italy; 9 Department of Medical and Surgical Specialties, Radiological Sciences and Public Health, University of Brescia, Brescia, Italy; 10 Department of Intensive Care and Anesthesiology, Fondazione Policlinico, Universitario A. Gemelli, Roma, Italy

**Keywords:** Artificial respiration, Positive-pressure respiration, Intensive care units, Patient-ventilator interaction, Inspiratory effort

## Abstract

**Background:**

The evaluation of patient effort is pivotal during pressure support ventilation, but a non-invasive, continuous, quantitative method to assess patient inspiratory effort is still lacking. We hypothesized that the concavity of the inspiratory flow-time waveform could be useful to estimate patient’s inspiratory effort. The purpose of this study was to assess whether the shape of the inspiratory flow, as quantified by a numeric indicator, could be associated with inspiratory effort during pressure support ventilation.

**Methods:**

Twenty-four patients in pressure support ventilation were enrolled. A mathematical relationship describing the decay pattern of the inspiratory flow profile was developed. The parameter hypothesized to estimate effort was named *Flow Index*. Esophageal pressure, airway pressure, airflow, and volume waveforms were recorded at three support levels (maximum, minimum and baseline). The association between *Flow Index* and reference measures of patient effort (pressure time product and pressure generated by respiratory muscles) was evaluated using linear mixed effects models adjusted for tidal volume, respiratory rate and respiratory rate/tidal volume.

**Results:**

*Flow Index* was different at the three pressure support levels and all group comparisons were statistically significant. In all tested models, *Flow Index* was independently associated with patient effort (*p* < 0.001). *Flow Index* prediction of inspiratory effort agreed with esophageal pressure-based methods.

**Conclusions:**

*Flow Index* is associated with patient inspiratory effort during pressure support ventilation, and may provide potentially useful information for setting inspiratory support and monitoring patient-ventilator interactions.

**Supplementary Information:**

The online version contains supplementary material available at 10.1186/s13054-021-03624-3.

## Background

During pressure support ventilation (PSV), assessing patient inspiratory effort could allow the titration of respiratory assistance in order to minimize over- and under-assistance [1–3]. In fact, during under-assistance, a vigorous spontaneous effort may generate excessive diaphragmatic loading and muscle injury, as well as regional lung injury due to stress and strain [4–7]. Over-assistance, on the other hand, may lead to pulmonary hyperinflation and diaphragmatic atrophy and dysfunction, thus impairing weaning and prolonging the dependency from mechanical ventilation [1, 2, 8, 9]. Independently from the appropriateness of inspiratory assistance, the patient's neuro-ventilatory drive and hence the breathing effort may unpredictably vary over time due to pain, anxiety, delirium, sepsis, sedation or other pathological conditions [10].

Despite its perceived importance, however, the inspiratory effort is seldom monitored [11]. Qualitative clinical surrogates to infer patient effort include respiratory rate (RR), tidal volume (V_T_), rapid shallow breathing index (RSBI, calculated as respiratory rate over tidal volume) and the use of accessory muscles [3, 12, 13]. Esophageal pressure measurement is the reference technique used to quantify inspiratory effort, but it requires technical proficiency and the insertion of an esophageal catheter [14, 15]. Other methods include monitoring of the diaphragm’s electrical activity and ultrasound assessment of inspiratory diaphragmatic displacement and thickness. Both are associated with drawbacks, such as availability, costs, non-continuous monitoring and operator-dependency [16].

**Fig. 1:**
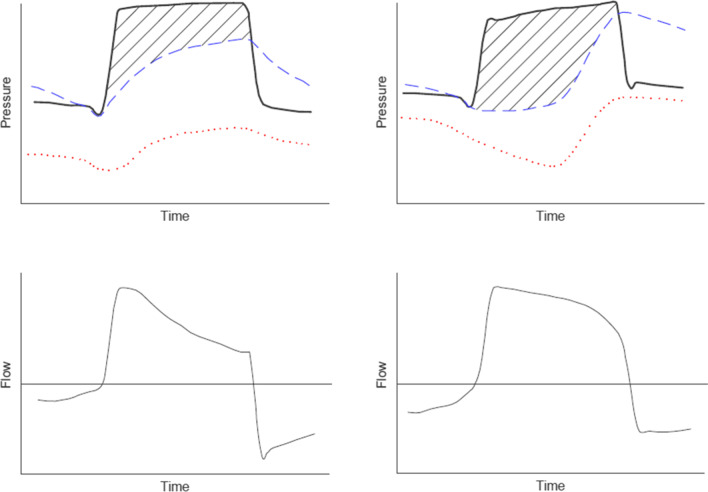
Physiologic rationale behind the use of the inspiratory flow curve to detect patient inspiratory effort. The upper panels show the pressure–time curve of airway pressure (P_aw_, full line), esophageal pressure (P_es,_ dotted line) and alveolar pressure (P_alv_, dashed line) during pressure support ventilation. The lower panels show the flow-time curve during pressure support ventilation. Left Panels: In absence of patient inspiratory effort, the inspiratory flow is maximal at the beginning of inspiration and the pressure gradient between the airway opening and the alveoli (*area with diagonal lines*) is progressively reduced. Thus, the inspiratory flow parallels the exponential decay of the airway opening-alveoli pressure difference. Right Panels: In presence of patient inspiratory effort, the fall in pleural pressure reduces alveolar pressure. The greater the inspiratory effort, the more the airway-alveolar pressure gradient is sustained *(area with diagonal lines)*. The inspiratory flow curve moves further away from the exponential decay towards a downward concavity shape, proportionally to the level of activation of inspiratory muscles

During PSV, the inspiratory portion of the flow-time waveform may provide important information regarding patient effort. Deviations of inspiratory flow from the exponential decay pattern that is typical of the passive patient ventilated in pressumetric ventilation, suggest that the patient is active during inspiration [17, 18], as shown in Fig. 1. However, to our knowledge, the quantitative relationship between the inspiratory flow-time waveform and the inspiratory effort at the bedside has not been formally evaluated. In this study, we hypothesized that, during PSV, the concavity of the inspiratory flow-time waveform can be quantified by a numeric indicator, named *Flow Index,* similarly to the *Stress Index* on airway opening pressure [19]. Aim of our study was to assess if *Flow Index* was associated with patient inspiratory effort during PSV.

## Methods

### Patients

This study was conducted in the Intensive Care Unit (ICU) of Fondazione Poliambulanza, Brescia, Italy. Patients were included if they met all of the following criteria: age > 18 years, dependence on invasive mechanical ventilation (i.e. not ready to wean or having failed a spontaneous breathing trial on the day of the study [20]), being in PSV, having an esophageal balloon catheter already in place. Exclusion criteria were: mean arterial pressure < 60 mmHg, systolic arterial pressure > 180 mmHg, heart rate < 40/min or > 150/min, PaO_2_/FIO_2_ < 150 mmHg, pH < 7.35 with PaCO_2_ > 45 mmHg, diagnosis of head injury, intracranial hemorrhage or cerebral ischemia.

The protocol was approved by the local ethical committee (Comitato Etico della Provincia di Brescia). Written informed consent was obtained from the patient or, if the patients themselves were not competent, from their next of kin.

### Study protocol

The pressure support (PS) level at patient enrollment was defined as *baseline PS* (PS_base_). In order to explore a wide range of PS assistance, maximal and minimal PS (PS_max_ and PS_min_, respectively) were titrated. The PS_max_ was sought by progressively increasing the PS level until all signs of inspiratory muscle activity disappeared after inspiratory triggering, as assessed by visual inspection of the waveform of esophageal pressure (P_es_), airway opening pressure (P_aw_) and airflow. In order to avoid lung injury, the peak airway pressure was limited to a maximum of 30 cmH_2_O, regardless of achieving complete absence of inspiratory muscle activity. The PS_min_ was identified as the lowest PS level without dyspnea, keeping the RSBI < 100 min^−1^ l^−1^. Apart from the PS level, all the remaining ventilation variables (FIO_2_, inspiratory trigger, expiratory trigger) remained constant, as previously set by the attending physician. PS_base_, PS_min_ and PS_max_ were delivered in random order to every patient for 20 min each. PS levels were applied without interruption, and the recordings were obtained after letting the patient adapt for 20 min to each new PS level.

### Measurements and calculations

*Pes* was measured by an esophageal balloon catheter (Marquat Gbm, Boissy-St-Léger Cedex, France) connected to a pressure transducer (AS3/CS3; Datex-Engstrom Division, Instrumentarium Corp., Helsinki, Finland). The esophageal balloon was introduced 40 cm from the nostril and inflated with 1 ml of air. The occlusion test was used to assess if the P_es_ was appropriately transduced [21, 22]. The position of the balloon in the esophagus and its filling volume were modified, if necessary, so as to obtain a ratio between P_es_ and P_aw_ swings ranging between 0.8 and 1.2 during the occlusion [23, 24].

P_aw_, airflow, volume and P_es_ waveforms were recorded for each PS level for 5 min at the sampling rate of 100 Hz (Datex-Ohmeda S/5 Collect; Datex-Ohmeda Division, Instrumentarium Corp., Helsinki, Finland). Mean tidal volume (V_T_) and respiratory rate (RR) were also obtained for each patient at the 3 levels of PS. The RSBI was calculated as RR/V_T_.

All measurements were obtained from the longest portion of the esophageal pressure waveform without swallowing artifacts (detected by a transient, sudden increase on the pressure trace), recorded at the end of the 20 min at each PS level.

The onset of inspiration was identified by the first positive value in the flow recording, while the end of inspiration was identified by the last positive value of the inspiratory flow. The static recoil pressure of the chest wall (P_cw_) was calculated as the product of the inspired V_T_ and the measured chest wall elastance (E_cw_). E_cw_ was calculated as the ratio between the inspiratory change in P_es_ [end-inspiratory plateau esophageal pressure (P_plat,es_) minus end-expiratory plateau esophageal pressure (P_exp,es_)] and V_T_ obtained during PS_max_, i.e. in a condition of near relaxation.

**Fig. 2:**
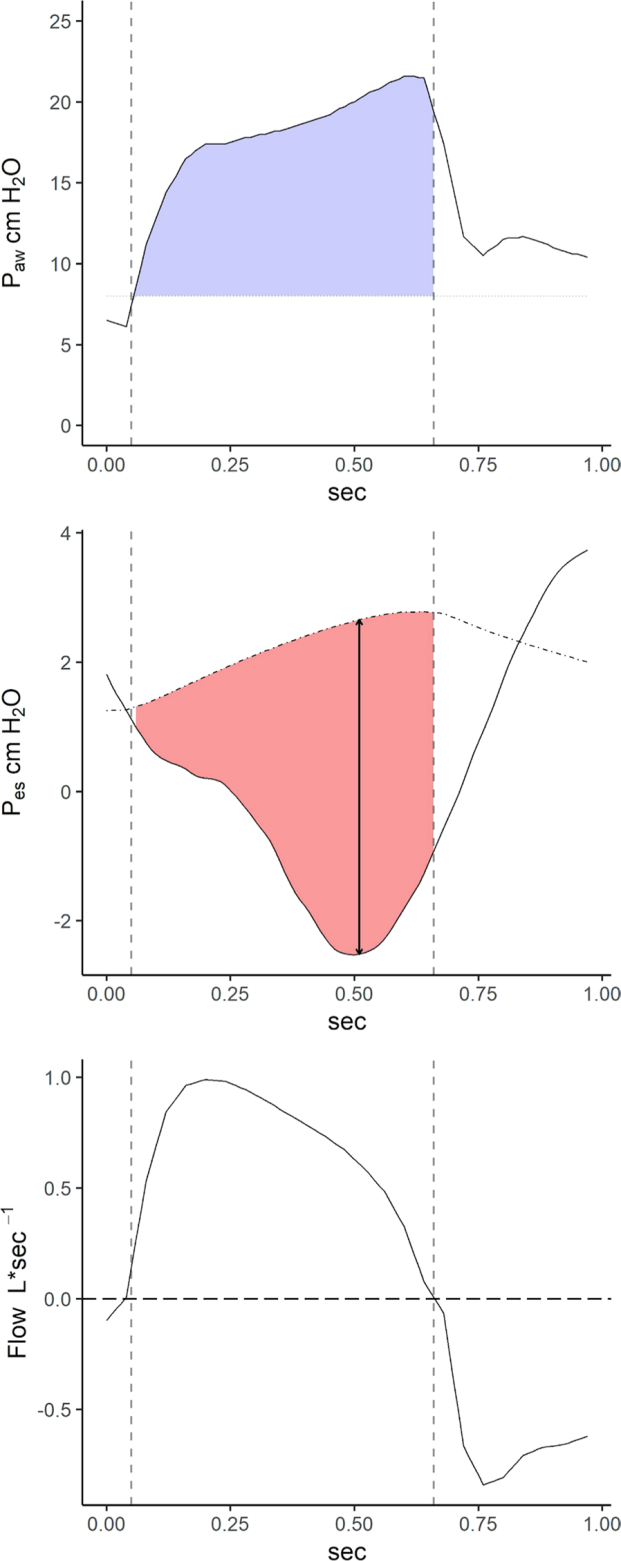
Airway pressure (P_aw_), esophageal pressure (P_es_) and airflow curves from a study participant. Upper panel: airway pressure (P_aw_) curve. The inspiratory phase is identified by dashed vertical lines, while the Pressure Time Product of the ventilator (PTP_vent_) is identified by the blue area, that is calculated as the product of inspiratory pressure (P_aw_ minus positive end expiratory pressure) for the duration of inspiration. Middle panel: esophageal pressure (P_es_) curve. The chest wall elastic recoil pressure (P_cw_) is identified by a dot-dashed line, while the Pressure Time Product of the patient (PTP_pt,breath_) is identified by the red area calculated as the product of P_cw_ minus P_es_ for the duration of inspiration. The maximum pressure generated by respiratory muscles (P_musc_) is identified by the arrow. Lower panel: inspiratory flow trace

The pressure generated by inspiratory muscles (P_musc_) was calculated as the maximal difference between P_cw_ and P_es_ (Fig. 2). The Pressure–Time Product of a single breath (PTP_pt,breath_) was calculated as the area between P_cw_ and P_es_ during tidal inspiration (Fig. 2). Similarly, the PTP of the mechanical ventilator for each breath (PTP_vent,breath_) was obtained as the area between the P_aw_ and the set PEEP during inspiration. The total PTP (PTP_tot, breath_) was the sum of PTP_pt,breath_ and PTP_vent,breath_. The fraction of PTP generated by the patient during pressure support ventilation (PTP_ratio,breath)_ was calculated as follows:

1 $${\text{PTP}}_{{\text{ratio,breath}}} = {\text{PTP}}_{{\text{pt,breath}}} /{\text{PTP}}_{{\text{tot,breath}}}$$

### The *Flow Index*

**Fig. 3:**
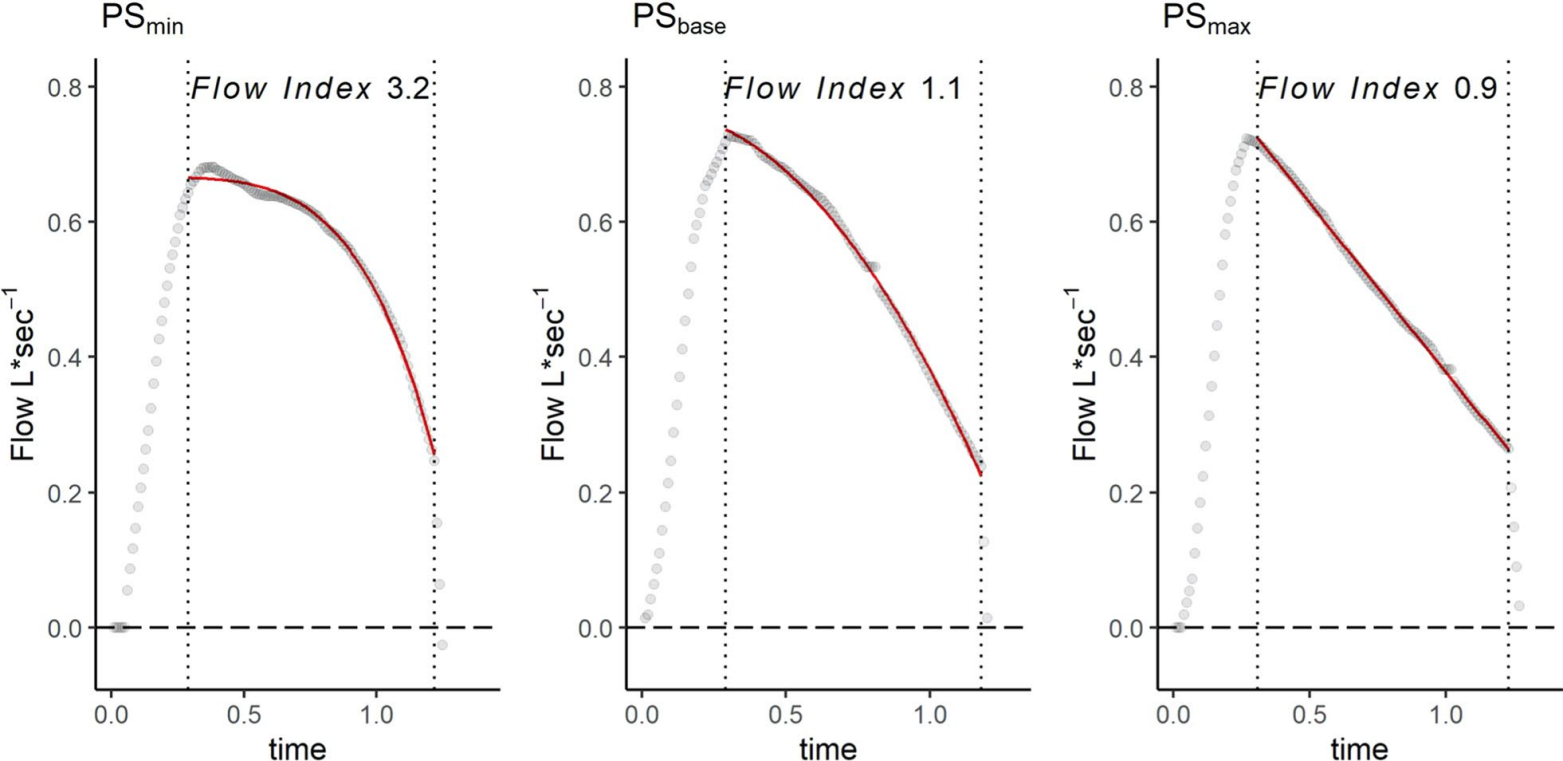
Procedure used to calculate *Flow Index* from the descending inspiratory portion of the flow waveform. Flow waveforms at the three different pressure support levels. The grey circles indicate the sampled inspiratory flow values, while the vertical lines indicate the cutting points used to select the descending inspiratory portion of the flow waveform. The red line shows the fitted model, calculated using Eq. 2 (detailed explanation in text). PS_min_ = minimum pressure support, PS_base_ = baseline pressure support, PS_max_ = maximum pressure support

The portion of the inspiratory flow-time waveform included between the end of the ramp and before the expiratory trigger was used to calculate the *Flow Index* (Fig. 3). A non-linear model, described by Eq. 2, was fitted to this portion of the flow waveform for every breath analyzed.

2 $$\dot{V} = a + {\text{b}} \cdot \Delta \;{\text{time}^c}$$

In this model, the inspiratory flow ($\dot{V}$) is a function of time, of peak flow (*A*), of the rate of flow reduction (*b*) and of parameter *c*, which describes the downward facing concavity of the portion of the inspiratory flow waveform. The parameter *c*, calculated for every breath, was named *Flow Index*. The inspiratory flow waveform was automatically cut at two different points, in order to select its descending portion and estimate the parameter *c* or *Flow Index* (Fig. 3). The initial cut point was selected by excluding all the measurements in which the flow increased more than 1% of the preceding measurement, meaning that flow trace quickly increased towards its peak with the rate of increase depending on the ramp. The final cut point was identified as the point where the measured flow decreased more than 10% of the preceding measurement, indicating the beginning of the cycling toward expiration. The portion of the flow trace between the initial and the final cut point was used to estimate the *Flow Index* by fitting a non-linear model, as described by Eq. 2.

The *Flow Index* describes the concavity of the curve using the same equation that computes the well-known *Stress Index*, a visual and quantitative parameter of overdistension detectable from the inspiratory airway opening pressure profile during constant-flow controlled mechanical ventilation [19, 25, 26]. The *Flow Index* is equal to 1 when the inspiratory flow decreases linearly. If the waveform has an upward facing concavity, the *Flow Index* is < 1, whereas if the curve has a downward facing concavity, the *Flow Index* is > 1. The principle supporting visual inspection of the inspiratory decay of the airflow waveform to estimate patient effort has been previously suggested [17, 18]. The *Flow Index* rationale arises from the analysis of the inspiratory flow during pressometric mechanical ventilation in the passive patient, which is characterized by constant pressure applied to the airway opening throughout the entire inspiratory time. The inspiratory flow is driven by the difference of pressure between the airway opening and alveoli, which is maximal at the beginning of inspiration when the alveoli have their minimal pressure (Fig. 1). Thus, in absence of patient inspiratory effort, the inspiratory flow is maximal at the beginning of inspiration. If a patient remains passive throughout the inspiration, alveolar pressure exponentially increases as the volume fills the alveoli, proportional to the elastance of the respiratory system. This progressively decreases the pressure gradient between the airway opening and the alveoli, and inspiratory airflow parallels the exponential decay of the opening airway-alveoli pressure difference. When a patient activates the inspiratory muscles during the inspiratory phase, the fall in pleural pressure tends to increase the chest wall and lung volume, decreasing the alveolar pressure. Thus, the greater the inspiratory effort, the more the gradient between airway opening and alveolar pressure will be sustained. This mechanism increases the instantaneous inspiratory flow as compared to the passive condition and modifies its shape profile, from an exponential decay towards a downward concavity (Fig. 1). We hypothesized that these changes in the inspiratory waveform profile, as quantified by the *Flow Index*, would be proportional to the activation of the inspiratory muscles and thus useful to quantify patient inspiratory effort.

### Outcomes

The primary outcome was the association between the *Flow Index* and patient respiratory effort, as expressed by PTP_pt,breath_, PTP_ratio,breath_ and P_musc_.

### Statistical analysis

We planned to assess sample size after enrolling the first 20 patients. A power analysis was carried out using Montecarlo simulation, employing a linear mixed model with a random effect for each patient. Enrolling 24 patients would grant a power of 85% with alpha = 0.05 to detect an effect size of 0.10 cmH_2_O⋅s^−1^ increment in PTP_pt,breath_ for each unitary increase in *Flow Index*.

Data are shown as mean ± standard deviation, median (1st–3rd quartile) or frequency (percentage), as appropriate. Variables at the three levels of support were compared with analysis of variance (ANOVA) for repeated measure and post-hoc Tukey's honestly significant difference (HSD) test.

*Flow Index* was compared in the different quartiles of inspiratory effort estimates (PTP_pt,breath_, PTP_ratio,breath_ and P_musc_) with ANOVA for repeated measures and post-hoc Tukey's HSD test.

Linear mixed effects models were used to assess the association between *Flow Index* and PTP_pt,breath_, PTP_ratio,breath_ and P_musc_, both unadjusted and adjusted for RR and V_T_, with patients managed as random effects. These models were compared with a likelihood ratio test to a model including *Flow Index,* to assess if *Flow Index* improved the goodness of fit [27]. Marginal R^2^ and conditional R^2^ were computed, expressing the amount of variance in the dependent variable which could be explained by the mixed models, respectively excluding and including the variance explained by the random effect in the models [28].

In order to explore the association between Flow index and the PTP_pt,breath_ for every single patient, we estimated 24 linear models, one for each patient (see Additional file 1: Fig. S1).

A *p* value lower than 0.05 was considered significant. Statistical analyses were performed with R (R Core Team, 2018. R Foundation for Statistical Computing, Vienna, Austria) with packages “lme4” (version 1.1-21), “multcomp” (version 1.4-11).

## Results

A total of 24 patients were included in the study. Patients’ characteristics are shown in Table 1. Breathing pattern, levels of inspiratory effort and *Flow Index* at the three levels of PS are shown in Table 2. The increase in PS level was associated with higher V_T_ and lower RR, with similar minute ventilation. The level of inspiratory effort significantly decreased by raising PS levels.

**Table 1:** Patients’ characteristics

Age (years)	74 ± 9.7
Female, *n* (%)	6 (25)
Body Mass Index (kg m^−2^)	26.7 ± 6.7
Height (cm)	168 ± 8.6
Days on mechanical ventilation at enrollment	9 (3–21)
Patients with tracheostomy on study day, *n* (%)	7 (30)
PEEP (cmH_2_O)	6 ± 1
FIO_2_	0.4 ± 0.08
pH	7.46 ± 0.04
PaCO_2_ (mmHg)	38 ± 5
PaO_2_ (mmHg)	88 ± 25
Hospital mortality, *n* (%)	4 (16%)
Length of stay in Intensive Care Unit (days)	25 (15–35)
Diagnosis	
ARDS	12 (50%)
COPD exacerbation	5 (21%)
Sepsis	10 (42%)
Trauma	3 (13%)

Data are shown as mean (standard deviation) or count (%). Measurements were taken at the time of patient enrollment

*PEEP* positive end expiratory pressure, *ARDS* acute respiratory distress syndrome, *COPD* chronic obstructive pulmonary disease

**Table 2:** Inspiratory effort and breathing pattern at PS_min_, PS_base_ and PS_max_

	PS_min_	PS_base_	PS_max_	*P* value
Set pressure support level (cmH_2_O)	4 ± 1	10 ± 3	18 ± 5	< 0.001
Tidal volume (ml)	440 ± 132	511 ± 146	671 ± 217	< 0.001
Tidal volume/IBW (ml kg^−1^)	6.6 (1.7)	7.9 (1.9)	10.5 (2.4)	< 0.001
Respiratory rate (min^−1^)	31 ± 10	26 ± 10	20 ± 6	< 0.001
RSBI	78 ± 36	58 ± 31	34 ± 19	< 0.001
Minute ventilation (l min^−1^)	11.6 ± 4.0	11.7 ± 4.3	11.7 ± 3.9	0.98
PTP_pt,minute_ (cmH_2_O⋅s min^−1^)	83 ± 93	32 ± 94	8 ± 26	< 0.001
PTP_pt,breath_ (cmH_2_O⋅s)	4.0 (2.9)	2.3 (2.2)	0.9 (1.4)	< 0.001
PTP_ratio,breath_	0.76 ± 0.3	0.29 ± 0.28	0.06 ± 0.09	< 0.001
P_musc_ (cmH_2_O)	7.5 (4.4)	4.3 (3.6)	1.9 (2.6)	< 0.001
*Flow Index*	4.6 ± 3.3	3.3 ± 2.5	1.9 ± 1.3	< 0.001

All pairwise comparisons between the three PS levels were significant (*p* < 0.05), except for minute ventilation

PS_min_ = minimal pressure support, PS_base_ = baseline pressure support, PS_max_ = maximal pressure support, RSBI = Rapid Shallow Breathing Index obtained by the ratio of respiratory rate and V_T_, PTP_pt,minute_ = Pressure–time product for the patient in one minute, PTP_pt,breath_ = Pressure–time product for the patient in a single breath, PTP_ratio,breath_ = PTP_pt,breath_/(PTP_pt, breath_ + PTP_vent, breath_), P_musc_ = pressure generated by respiratory muscles

### Association between *Flow Index* and inspiratory effort

**Fig. 4:**
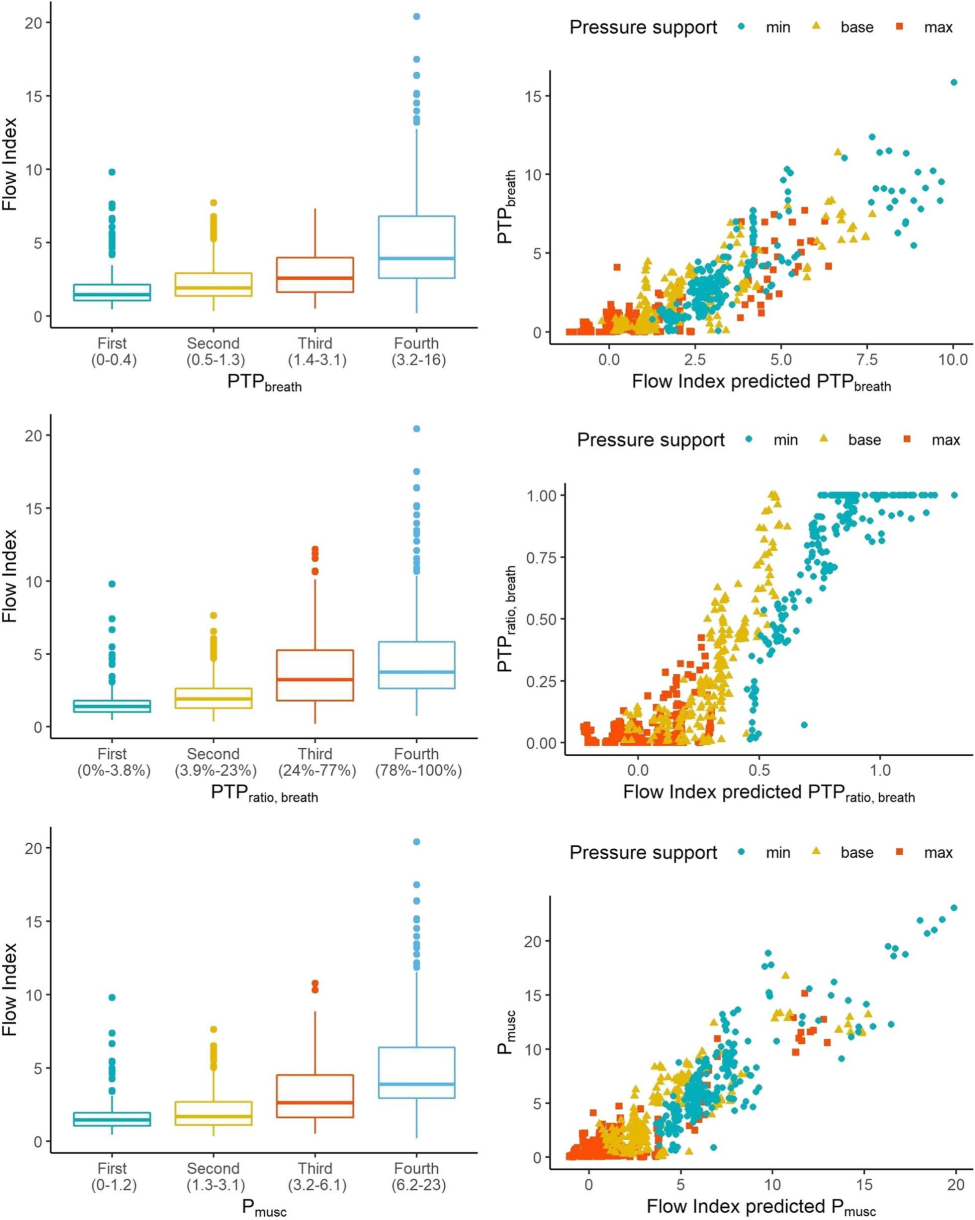
Box plot of quartiles of respiratory effort and *Flow Index.* Scatter plot of *Flow Index* model prediction and measured pressure time product, pressure time product ratio and muscular pressure. Left column: box plot of *Flow Index* in quartiles of inspiratory effort as evaluated with PTP_pt, breath_, PTP_ratio,breath_ and P_musc_. Right column: scatter plot of *Flow Index* model prediction for PTP_pt, breath_, PTP_ratio,breath_ and P_musc_ compared with measured PTP_pt, breath_, PTP_ratio,breath_ and P_musc_. Different PS levels are identified with different colors, as indicated above each panel. PTP = pressure time product

*Flow Index* was different at the three PS levels and all group comparisons were statistically significant at the post hoc analysis (PS_base_ vs. PS_min_ *p* = 0.005; PS_base_ vs. PS_max_ *p* < 0.001; PS_max_ vs. PS_min_ *p* < 0.001). Figure 4 shows, on the left side, the boxplots of *Flow Index* at different quartiles of PTP_pt,breath_, PTP_ratio,breath_ and P_musc_. All the comparisons were statistically significant (*p* < 0.01), except for those between the first and second quartile. On the right side, Fig. 4 shows the scatter plots of *Flow Index*-predicted against measured PTP_breath_, PTP_ratio,breath_ and P_musc_. Table 3 shows the coefficients of association resulting from the linear effects models between *Flow Index* and PTP_pt,breath_, PTP_ratio,breath_, P_musc_, both unadjusted and adjusted for RR and V_T_.

**Table 3:** *Flow Index* coefficient of unadjusted and adjusted linear mixed effects models

	*Flow Index* coefficient (95% CI)	*P* value	*Flow Index* coefficient (95% CI) adjusted for RR and V_T_	*P* value
PTP_ratio, breath_	0.03 (0.03, 0.04)	< 0.0001	0.03 (0.03,0.04)	< 0.0001
PTP_pt,breath_	0.19 (0.13, 0.25)	< 0.0001	0.23 (0.17, 0.29)	< 0.0001
P_musc_	0.34 (0.24, 0.43)	< 0.0001	0.33 (0.23 0.42)	< 0.0001

RR = respiratory rate, V_T_ = tidal volume, PTP_pt,breath_ = Pressure–time product for the patient in the single breath, PTP_ratio,breath_ = PTP_pt,breath_/(PTP_pt, breath_ + PTP_vent, breath_), P_musc_ = pressure generated by respiratory muscles

Moreover, in 22 out of 24 patients there was a positive and statistically significant association between Flow Index and the PTP_pt,breath_ for every single patient (see Additional file 1: Fig. S1).

### Comparison between models

The model fit significantly improved when the *Flow Index* was added to RR and V_T_ (p value for the likelihood ratio test < 0.001 for PTP_pt,breath_, PTP_ratio,breath_ and P_musc_), indicating that the *Flow Index* improved the inspiratory effort prediction.

The model for P_musc_ estimated an increase of 0.33 (95% CI 0.23, 0.42) cmH_2_O for each unitary increase in *Flow Index* (conditional *R*^2^ 0.77), the estimated increase in PTP_pt,breath_ was 0.23 (95% CI 0.17–0.29) cmH_2_O∙s^−1^ for each unitary increase in *Flow Index* (conditional *R*^2^ 0.7), while each unitary increase in *Flow Index* was associated with an increase of 3.2% (95% CI 2.5–3.9%) in PTP_ratio,breath_ (conditional *R*^2^ 0.86).

## Discussion

This study shows that *Flow Index* is independently associated with patient inspiratory effort and can give information on patient-ventilator interaction and on the distribution of respiratory effort between patient and ventilator.

We found that the *Flow Index* gives more insight on patient effort than the traditional analysis of the respiratory pattern based on the evaluation of RR and V_T_. This is a remarkable result since in clinical practice the inspiratory effort is seldom measured, and RR and V_T_ are deemed as useful surrogates for setting the PSV level [29]. Indeed, PSV level is usually titrated to obtain a VT between 5 and 8 ml/kg predicted body weight (PBW) and a RR between 20 and 30 breaths/min [6, 10]. The RSBI [13], which, by expressing an imbalance between load and effort, could be deemed as a sign of inadequate inspiratory support was not taken into account in the primary analysis since our model included both RR and V_T_. Nonetheless, we performed an unplanned post hoc supplementary analysis evaluating the association between Flow Index and RSBI. The results are similar to the ones we obtained for RR and VT.

The analysis of the respiratory waveforms is an essential bedside activity for intensivists but, to our knowledge, this is the first attempt to assess patient inspiratory effort based on the quantitative analysis of the inspiratory flow-time waveform, since all available evaluations of patient inspiratory effort are focused on the inspection of the pressure curve.

In this study the association between *Flow Index* and respiratory effort was assessed in a wide range of inspiratory efforts, as witnessed by the fact PT_pt,breath_ and P_musc_ were 400% higher at PS_min_ than at PS_max_. Since measuring the *Flow Index* is relatively simple, continuous and non-invasive, we suggest that this parameter could be suitable to titrate and monitor patient-ventilator interactions during PSV. However, this was a physiological study and further studies are needed to evaluate if the setting of PSV according to the *Flow Index* or monitoring the inspiratory effort during PSV over time could have an impact on clinically meaningful outcome parameters*.*

Since *Flow Index* is measured on a single breath, it was gauged against other single breath indicators of patient effort (PT_pt,breath_, P_musc_ and PT_ratio,breath_). Theoretically the best association with *Flow Index* could be with PT_ratio,breath_, since the latter depends on the interaction between support from the ventilator and patient effort. Nonetheless, our data show a strong association between *Flow Inde*x, PT_pt,breath_ and P_musc_. Accordingly, the *Flow Index* may be taken as a surrogate of the absolute patient inspiratory effort, as expressed with P_musc_ or PT_pt,breath_.

Several invasive and non-invasive estimates of patient effort were proposed in recent decades [3]. The pressure muscle index (PMI) and the least square fitting method (LSF) were among the first ones that were advanced. These techniques, however, required intermittent inspiratory occlusions or complex calculations, respectively, and their accuracy in evaluating single breath inspiratory effort was scarcely accurate [30–32]. Of note, PMI evaluates end-inspiratory effort only and not the overall effort during inspiration, since it does not take into account the inspiratory resistive load. A recent study showed a significant association between the negative swing in P_aw_ generated by respiratory muscle effort during assisted ventilation when the airway is briefly occluded (P_occl_) and patient inspiratory effort**.** Though this method is of interest, a manual occlusion is needed and P_occl_ could not provide continuous monitoring of patient inspiratory effort [11]. The assessment of diaphragmatic electrical activity (E*di*) is an accurate measure of neuro-ventilatory drive, and P_musc_ may be estimated through the calculation of neuromuscular efficiency, obtained by comparing E*di* and P_occl_ during an end-expiratory airway occlusion, or through the calculation of neuro-ventilatory efficiency, obtained by comparing E*di* and V_T_ [10, 33, 34]. However, measuring E*di* is costly, requires the insertion of a patent-protected nasogastric catheter which functions only with specific mechanical ventilators and, finally, a manual occlusion is required to calculate neuromuscular efficiency [33]. Moreover, during inspiratory effort the contribution of diaphragm and other inspiratory muscles may vary, since a great variability in diaphragm contribution to inspiration exists (as can be seen, for example, between male and female patients [34–36]). E*di* allows detection of the diaphragm contribution exclusively, and therefore it can lead to loss of important information for patients in whom the diaphragm contribution is low [33]. Diaphragm ultrasound has also been proposed as a tool to estimate patient effort, but it remains a punctual measure, dependent on the operator’s technical ability and heavily influenced by the degree of diaphragm dysfunction [37, 38].

Strengths of the *Flow Index* are its non-invasiveness, the possibility to monitor it continuously and the independence from occlusion maneuvers which is particularly advantageous since not all mechanical ventilators permit a manual expiratory occlusion during PSV [39, 40]. Since the flow waveform is displayed on the majority of mechanical ventilators, visual inspection of *Flow Index* by trained physicians could also give valuable information, albeit only qualitative, on inspiratory effort. More studies are needed to know if visual inspection of the flow waveform is sufficient to assess *Flow Index*, but since the *Stress Index* uses a similar equation and its visual inspection has been proved to be effective [25], the same may be applicable to *Flow Index*. Of note, the *Flow Index* algorithm could be easily implemented on standard monitoring, as has been done for the *Stress Index* [19], permitting a quantitative evaluation of patient effort during mechanical ventilation.

This study presents four main limitations. First, it is a single center study, and therefore its external validity needs to be proved by additional studies. Second, some portions of the inspiratory effort are not included in the calculation of Flow Index, since they occur before the peak inspiratory flow is reached. Despite this potential limitation, the Flow Index shows a good association with the post-triggering inspiratory effort. This could be due to the fact that the rise time usually lasts a really short time (less than 100 ms), and therefore has little impact on the patient’s overall inspiratory effort. Third, the *Flow Index* should be specifically assessed in patients with a high resistive load, in which a high respiratory effort may not correspond to a proportional increase in inspiratory flow. Of note, the *Flow Index* does not account for patient effort before the inspiratory flow, which should be considered in patients with severe dynamic hyperinflation. Fourth, our study does not give any cutoffs useful to detect high or low inspiratory effort, and further studies should assess this relevant aspect.

## Conclusions

*Flow Index* is a novel quantitative, continuous, non-invasive evaluation of the concavity of the inspiratory flow profile. Our study show that the *Flow Index* is associated with patient inspiratory effort during PSV and that it gives additional information when compared to the variables traditionally used for inspiratory effort evaluation during pressure support ventilation (i.e. V_T_, RR and RSBI). Additional research is needed to evaluate the potential impact of assessing the *Flow Index* on patient-ventilator interaction and weaning from mechanical ventilation.

## Supplementary Information

**Additional file 1**. Association between* Flow Index* and PTP_pt,breath_ for every single patient.

## Data Availability

The datasets during and/or analysed during the current study available from the corresponding author on reasonable request.

## Abbreviations

ANOVA: analysis of variance
E*di*: diaphragmatic electrical activity
E_cw_: measured chest wall elastance
HSD: honestly significant difference
ICU: Intensive Care Unit
LSF: least square fitting method
PSV: pressure support ventilation
PS: pressure support
PS_base_: baseline pressure support
PS_max_: maximal pressure support
PS_min_: minimal pressure support
P_es_: esophageal pressure
P_aw_: airway opening pressure
P_cw_: static recoil pressure of the chest wall
P_musc_: pressure generated by respiratory muscle
P_plat,es_: end-inspiratory plateau esophageal pressure
P_exp,es_: end-expiratory plateau esophageal pressure
PTP_pt,breath_: pressure–time product of a single breath
PTP_vent,breath_: pressure–time product of a mechanical ventilator for each breath
PEEP: positive end expiratory pressure
PTP_tot, breath_: total pressure–time product for each breath
PTP_ratio,breath_: fraction of pressure–time product generated by the patient for each breath
PMI: pressure muscle index
P_occl_: negative swing in P_aw_ generated by respiratory muscle effort during assisted ventilation when the airway is briefly occluded
RR: respiratory rate
RSBI: rapid shallow breathing index, calculated as respiratory rate over tidal volume
V_T_: tidal volume
$${\dot{V}}$$: inspiratory flow
