# Supplementary material for: Flow Index: a novel, non-invasive, continuous, quantitative method to evaluate patient inspiratory effort during pressure support ventilation
Source: Crit Care. 2021 Jun 7;25:196. doi: 10.1186/s13054-021-03624-3 (PMC8182360; doi:10.1186/s13054-021-03624-3)
Supplement: Supplementary file 1 — Additional file 1. Association between Flow Index and PTPpt,breath for every single patient. [file 13054_2021_3624_MOESM1_ESM.docx]

**Additional file 1**

**Figure S1. Association between Flow index and PTP_pt,breath_ for every single patient**


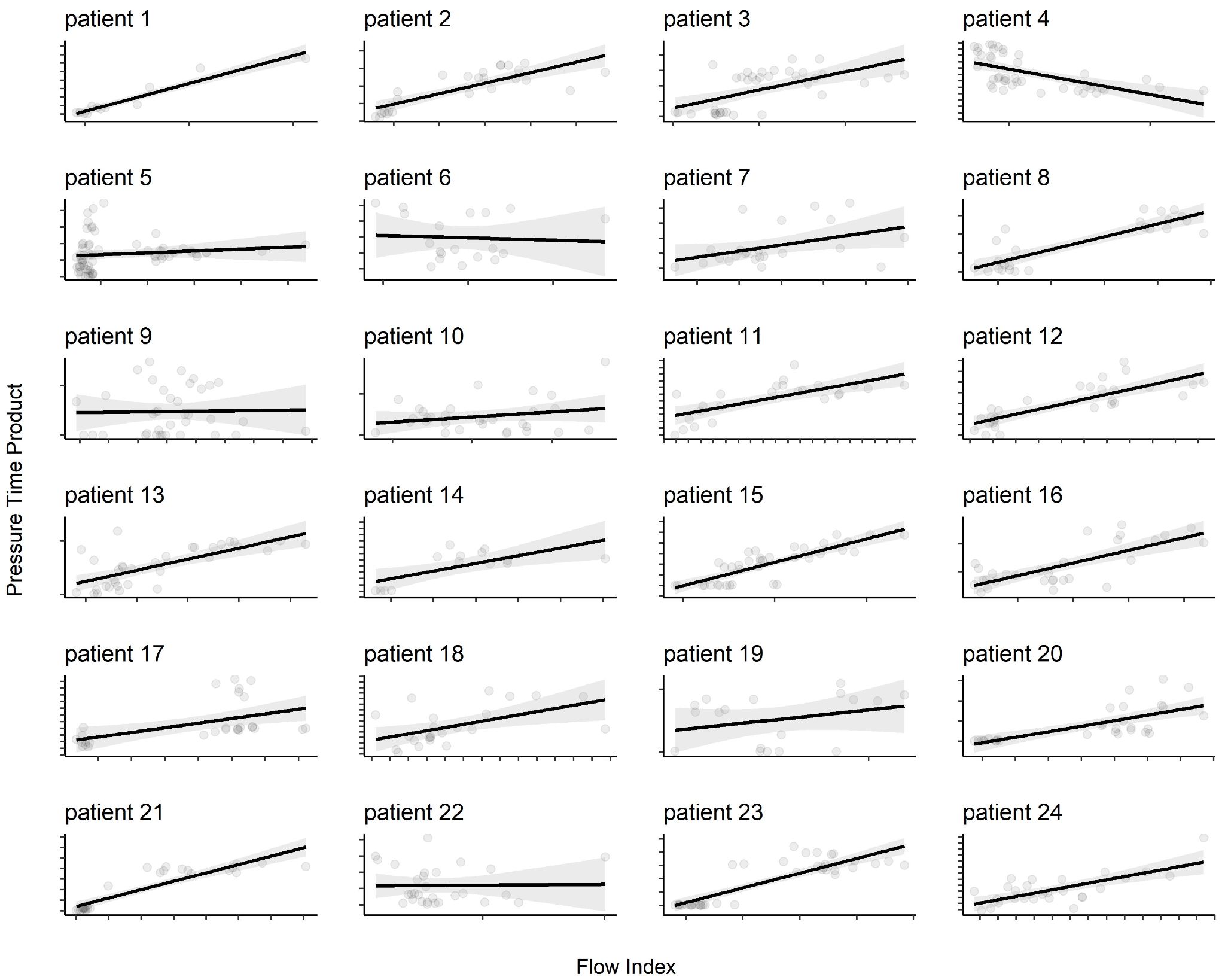


In order to explore the association between Flow index and the PTP_pt,breath_ for every single patient, we estimated 24 linear models, one for each patient. The coefficient for Flow Index was positive and statistically significant in 22 out of 24 patients, with the exception of patient 4 and 6 (see Figure), with value of 0.47 (0.19-0.72) cmH_2_O · sec^-1^ for PTP_pt,breath_. Abbreviations:

PTP_pt,breath_ = Pressure-Time Product of a single breath
